# Antiobesity and anti-inflammation effects of Hakka stir-fried tea of different storage years on high-fat diet-induced obese mice model via activating the AMPK/ACC/CPT1 pathway

**DOI:** 10.29219/fnr.v64.1681

**Published:** 2020-06-08

**Authors:** Qiuhua Li, Xingfei Lai, Lingli Sun, Junxi Cao, Caijin Ling, Wenji Zhang, Limin Xiang, Ruohong Chen, Dongli Li, Shili Sun

**Affiliations:** 1 Tea Research Institute, Guangdong Academy of Agricultural Sciences/Guangdong Provincial Key Laboratory of Tea Plant Resources Innovation & Utilization, Guangzhou, China; 2 School of Biotechnology and Health Sciences, Wuyi University, Jiangmen, China

**Keywords:** Hakka stir-fried tea, obesity, inflammation, fat accumulation, hepatic steatosis, AMPK/ACC/CPT1 pathway

## Abstract

**Background:**

As a typical representative of metabolic syndrome, obesity is also one of the extremely dangerous factors of cardiovascular diseases. Thus, the prevention and treatment of obesity has gradually become a global campaign. There have been many reports that green tea is effective in preventing obesity, but as a kind of green tea with regional characteristics, there have been no reports that Hakka stir-fried tea (HT) of different storage years has a weight loss effect.

**Aims:**

The aim was to investigate the effect of HT in diet-induced obese mice.

**Methods:**

The mice were divided into five groups as follows: the control group received normal diet; the obese model group received high-fat diet; and HT2003, HT2008, and HT2015 groups, after the induction of obesity via a high-fat diet, received HT of different storage years treatment for 6 weeks, respectively.

**Results:**

It was observed that HT decreased the levels of serum and liver triglyceride; the ratio of liver to body weight; accumulation of epididymal, perirenal, and mesenteric fat; the degree of hepatic steatosis; and adipocyte hypertrophy, with the concomitant reduction of body weight. Moreover, HT decreased the expression levels of proinflammatory cytokines tumor necrosis factor α (TNF α), inducible nitric oxide synthase (iNOS), cyclooxygenase-2 (COX-2) and reduced fatty acid synthase (FAS) activity in liver tissue of obese mice. In addition, HT treatment also increased the phosphorylation of AMP-activated protein kinase (AMPK) and its direct downstream proteins, acetyl coenzyme A carboxylase (ACC), and carnitine palmitoyltransferase I (CPT-1), which participate in FAS pathway.

**Conclusions:**

These findings demonstrate that HT treatment has a potential protection on high-fat diet-induced obesity mice via activating the AMPK/ACC/CPT1 pathway, and to a certain extent, it has nothing to do with the storage time of three kinds of HT.

## Popular scientific summary

- We confirmed that HT treatment significantly reduced body weight and fat accumulation in major organs in high-fat diet-induced obese mice.
- The effects of HT appear to be mediated by the activation of AMPK/ACC/CPT-1 pathway and further inhibiting lipogenesis.
- Our findings suggest that HT may be used as a potential dietary strategy for preventing obesity and related metabolic disorder diseases.

As we know, metabolic syndrome is defined as a general term for a range of cardiovascular disease risk factors (1). The accumulation of these factors in the same individual significantly increases the probability of cardiovascular disease, among which central obesity, hyperglycemia, hypertriglyceridemia, and hypertension are called the death quartet of metabolic syndrome (2–5). As one of the most dangerous factors inducing cardio vascular disease (CVD), obesity has reached a global and challenging stage not only in industrialized countries, but has radiated its effect to developing countries, including China (6, 7). According to the results of China’s National Nutrition and Health Survey over the years, the high economic burden of overweight and obesity suggests an urgent need to develop effective interventions to control obesity epidemics and thus prevent chronic diseases (8). Therefore, the prevention and treatment of obesity has gradually become a nationwide campaign.

The etiology of obesity is complex; genetic characteristics, sedentary lifestyle, dietary factors, particularly the indulgence in high-fat diet, are considered as major risk factors for its development (9). The consumption of high-fat diet induces obesity, characterized by a more positive energy balance, leading visceral and abdominal fat deposition finally (10). A large number of clinical data studies have shown that a great part of therapeutic regimens used for the long-term weight loss are largely ineffective, where 90–95% of people who lose weight subsequently regain it. Some antiobesity drugs for obesity treatment, such as orlistat, rimonabant, and sibutramine, have therapeutic effects mainly by affecting the lipid mobilization and utilization (11). However, owing to the adverse side effects associated with many traditional antiobesity drugs, more recent researches have shifted the focus to screening natural drugs with fewer side effects. This will provide an excellent alternative strategy for developing safer and more efficient antiobesity drugs in the future.

Tea (*Camellia sinensis*) originated in China and has a long history. After thousands of years of development and dissemination, tea has become one of the most popular beverages in the modern world, second only to water (12). Depending on the difference of production method and fermentation process, tea can be roughly defined as three types: the ‘non-fermented’ green tea, the ‘semi-fermented’ oolong tea, and the ‘fully fermented’ black/red tea (13). Obviously, different production processes lead to different chemical compositions, mainly reflected in the differences in the biological properties of different types of tea extracts (14). Green tea has only undergone minimal fermentation, and therefore contains predominately catechins (15). It is estimated that a typical brewed green tea beverage (1 g green tea in 100 mL boiling water) usually contains approximately 96–128 mg catechins, including epigallocatechin (EGC), catechin (C), epicatechin (EC), epigallocatechin gallate (EGCG), gallocatechin gallate (GCG), and epicatechin gallate (ECG) (16, 17). Among them, EGCG is the most abundant catechin present in green tea, accounting approximately for 30–50% of the total catechins (TC) content (18). In addition to the catechin constituents, green tea also contains quite a few of caffeine. Although it has been well documented that green tea, mainly active catechins, could exhibit antiobesity, hypolipidemic, and hypoglycaemic properties and thereby improving CVD, caffeine in green tea has the opposite effect on CVD (19, 20). Furthermore, caffeine not only has various negative effects on human behavior and sleep, but also causes great dilemma for those who are obese or diabetic (21). Thus, there is an urge for the market to search for green tea with low or no caffeine which could pose similar health benefits.

Hakka stir-fried tea (HT), as a typical representative of Chinese local green tea, is characterized by sweet and unbitter taste, stir-fried rice flavor, or sweet fragrance of flowers. There have been many reports that green tea is effective in preventing obesity. The mechanism of action is mainly concentrated in decreased lipid absorption, downregulated inflammation level, lowered lipid per-oxidation, and regulated glucose uptake system in adipose tissue and skeletal muscle (22–24). However, it remains to be seen whether storage changes the active components and the harmful caffeine in green tea. And the study on the effect of green tea in different storage years on weight loss has not been reported in detail. This study was to investigate the effects of Hakka stir-fried green tea after storage in different years (2003, 2008, and 2015) on the body weight and white fat content of obese mice induced by high-fat diet, and explore the molecular mechanism related to fat synthesis, revealing the mechanism of weight loss of Hakka green tea, providing theoretical basis for product development and active basic research of stir-fried green tea.

## Materials and methods

### Preparation of freeze-dried powder for tea aqueous extract

Three kinds of HT produced in 2003, 2008, and 2015 were all obtained from Tea Research Institute, Guangdong Academy of Agricultural Sciences in China. For the aqueous extracts preparation, three kinds of tea (100 g) were weighed, crushed, and ground, and then added into boiling distilled water (2,000 g) for 30 min at a ratio of 1:20 (W/V). Then, the tea soup was strained while it was hot, and this step was repeated three times. Last, the tea soup was concentrated by rotary evaporation to 1/5 of the original volume, and freeze-dried to obtain Hakka fried tea lyophilized powder.

### Effective component analysis of tea aqueous extract

The total amount of free amino acids (TFAA, standard number: GB/T 8314-2013) and the total amount of total tea polyphenols (TTP, standard number: GB/T 8313-2008) in the freeze-dried powder were detected by the implementation standards issued by the State Administration for Quality Supervision and Inspection and Quarantine in China, respectively. In addition, the total amount of soluble carbohydrates (TSC) was detected by anthrone-sulfuric acid colorimetric method and the total amount of flavonoids (TF) was determined by aluminum chloride (AlCl_3_) colorimetric method. For the content of different catechin monomers and caffeine, high-performance liquid chromatography (HPLC) method was used to test them. In brief, sample solution was injected onto a ZORBAX Eclipse XDB-C18 HPLC column (4.6 × 150 mm, 5 µm) (Agilent, Inc., CA, USA). A gradient elution was carried out using the following solvent systems: mobile phase A – double distilled water/phosphoric acid/acetonitrile/acetic acid (96.5/2/1/0.5; v/v/v/v); mobile phase B – double distilled water/phosphoric acid/acetonitrile/acetic acid (69.5/20/10/0.5; v/v/v/v). The elution was performed with a gradient procedure as follows: 0–30 min, from 72.5% A to 20% A, from 27.5% B to 80% B; 30–40 min, from 20% A to 72.5% A, from 80% B to 27.5% B. The column heater was kept at 28°C. The flow rate used was 1.0 mL/min, and the detection wavelength was performed at 280 nm. Each sample (10 µL) was injected into the column after filtration through a 0.45-µm filter disk. Identification of the different catechin monomers and caffeine was carried out by comparing the retention times and the UV absorbance of the unknown peaks to those of the standards. At the same time, a standard mixture containing caffeine (CAFF), gallic acid (GA), gallocatechin (GC), EGC, C, EC, EGCG, GCG, and ECG in methanol was prepared and analyzed. The correlative data were collected, integrated, and analyzed after standard curve calibration by the system. The TC content is equal to the sum of each monomer. Details of each active ingredient are shown in Table 1.

**Table 1:** Analysis of active components of Hakka stir-fried tea from three different storage years

Active ingredients/Group	Hakka stir-fried tea in 2003 (HT2003)	Hakka stir-fried tea in 2008 (HT2008)	Hakka stir-fried tea in 2015 (HT2015)
Total amount of free amino acids (TFAA) (%)	5.85 ± 0.01	4.97 ± 0.05^&&^	5.78 ± 0.02^$$^
Total tea polyphenols (TTP) (%)	37.05 ± 0.16	41.66 ± 0.40^&&^	45.10 ± 0.09^&&^^$$^
Total amount of soluble carbohydrates (TSC) (%)	10.92 ± 0.01	11.26 ± 0.06^&&^	10.22 ± 0.09^&&^^$$^
Total amount of flavonoids (TF) mg/mL	30.77 ± 0.08	28.97 ± 0.01^&&^	29.59 ± 0.01^&^
Caffeine (CAFF) (%)	5.78 ± 0.11	5.07 ± 0.11^&^	5.99 ± 0.36^$$^
Gallic acid (GA) (%)	0.18 ± 0.03	0.17 ± 0.00	0.24 ± 0.04
Gallocatechin (GC) (%)	1.22 ± 0.15	1.28 ± 0.04	1.96 ± 0.17^&&^^$$^
Epigallocatechin (EGC) (%)	0.34 ± 0.03	0.48 ± 0.01^&^	0.72 ± 0.07^&&^^$$^
Catechin (C) (%)	0.40 ± 0.05	0.53 ± 0.05^&^	0.71 ± 0.03^&&^^$$^
Epicatechin (EC) (%)	1.16 ± 0.15	1.35 ± 0.02	1.99 ± 0.17^&&^^$$^
Epigallocatechin gallate (EGCG) (%)	10.41 ± 0.15	11.34 ± 0.31^&&^	13.22 ± 0.03^&&^^$$^
Gallocatechin gallate (GCG) (%)	0.92 ± 0.19	0.89 ± 0.04	1.45 ± 0.19^&^^$^
Epicatechin gallate (ECG) (%)	2.13 ± 0.34	2.10 ± 0.18	2.63 ± 0.41
Total catechins (TC) (%)	16.77 ± 1.04	18.13 ± 0.56	22.93 ± 1.03^&&^^$$^

Note: Values represent means ± SEM (*n* = 3).

& *P* < 0.05

&& *P* < 0.01, compared with HT2003

$ *P* < 0.05

$$ *P* < 0.01, compared with HT2008.

### Animals and high-fat diet-induced obesity model

Sixty male specific pathogen-free C57BL/6 mice (7-week-old, 22 ± 2 g) were obtained from Beijing Huafukang Bioscience Co. Ltd., China. All mice were maintained under conditions of controlled temperature (22 ± 1°C) and humidity (60 ± 15%) in a 12-h light/dark cycle, with free access to drink the deionized water and fed the irradiated disinfectant basic feed. After 1 week of acclimatization, they were randomly divided into two groups: 1) normal chow-fed group (Control, *n* = 10) that received normal chow diet; 2) high-fat fed group (Model, *n* = 50) that received a high-fat diet containing additional 10% lard, 10% egg yolk powder, 1% cholesterol, 0.2% bile salt, 0.4% calcium hydrogen phosphate, and 0.3% stone powder on the basis of 78.1% basic feed. Both the basic feed and the high-fat feed were processed and prepared by Guangdong Medical Laboratory Animal Center. After 8 weeks of feeding, the model mice with an average weight gain of 20% or more than the control group were defined as successful. Finally, we selected 27 successful obesity mice and eight control mice for subsequent intervention experiments.

### Animal regrouping and drug intervention

After successful obesity model building, the selected mice were divided into five groups: 1) control group (*n* = 8), each mouse was given 10 µL/d distilled water by gavage while continuing to feed on the basic feed; 2) model group (*n* = 6), each mouse was given 10 µL/d distilled water by gavage while continuing to feed on the high-fat feed; 3) 2003 Hakka tea treatment group (HT2003, *n* = 7); 4) 2008 Hakka tea treatment group (HT2008, *n* = 7); and 5) 2015 Hakka tea treatment group (HT2015, *n* = 7). The mice in different drug intervention groups were given 10 µL/d (1 g/kg) aqueous solution of Hakka tea freeze-dried powder by gavage while continuing to feed on the high-fat feed. The mice body weights were measured once a week. At the end of the experiment, the body weight gain of each group of mice was calculated.

### Tissue processing

After 6 weeks of intervention, the mice were anaesthetized by 40 mg/kg pentobarbital (i.p.) after a 16-h overnight fast and whole blood was withdrawn by cardiac puncture. Blood was collected using heparin containing tubes, and serum was separated by centrifugation (3,000 rpm, 10 min). Serum were used immediately for lipid measurement or frozen at -80°C for future detection. Livers were immediately excised, weighed, and divided into smaller pieces for storage at -80°C (for molecular detection) or in 4% paraformaldehyde for histological analysis. In addition, epididymal, perirenal, and mesenteric fat pads were excised, weighed, and photographed.

### Biochemical analyses of serum and liver

The level of triglycerides (TG) (Product code: A110-1, Nanjing Jiancheng Bioengineering Institute) in serum was tested according to the corresponding kit instructions. For liver tissues, the same method as above was used to detect the levels of TG in the supernatant of tissue homogenate.

### Hematoxylin and eosin staining and analysis of liver and adipose tissue

A portion of the liver tissue and the mesenteric adipose tissue were fixed in 10% buffered formalin for at least 24 h, dehydrated with a sequence of ethanol solutions, and processed for embedding in paraffin. Sections of 5 μm in thickness were cut, deparaffinized, rehydrated, stained with hematoxylin and eosin (H&E), and examined under an Olympus BX-53 microscope (Olympus, Tokyo, Japan). The area of each adipocyte was quantified using Image J software (National Institutes of Health, Bethesda, MD).

### Protein extraction and Western blotting analysis

The total proteins of liver tissue and the inguinal adipose tissue in each group mice were extracted using total protein extraction kit (Jiancheng Bioengineering Institute, Nanjing, China) and quantified by the total protein assay kit (bicinchonininc acid [BCA] method). With that, whole proteins were electrophoresed under the conditions in 10–15% polyacrylamide gels. The separated proteins were transferred to polyvinylidene difluoride membranes. Nonspecific binding was blocked with 5% skimmed milk for 2 h at 37°C. And then, the membranes were incubated with rabbit anti-AMPK alpha (phosphor T172) (Product code: 2531, 1:1000, Cell Signaling Technology, Danvers, MA, USA), rabbit anti-AMPK alpha (Product code: 2532, 1:1000, Cell Signaling Technology, Danvers, MA, USA), rabbit anti-ACC (Product code: 3662S, 1:500, Cell Signaling Technology, MA, USA), rabbit anti-phospho ACC (Ser79) (Product code: 3661S, 1:2000, Cell Signaling Technology, MA, USA), rabbit anti-FAS (Product code: 3189, 1:1000, Cell Signaling Technology, MA, USA), rabbit anti-CPT1A (Product code: 12252, 1:1000, Cell Signaling Technology, MA, USA), rabbit anti-TNFα (Product code: ab6671, 1:2000, Abcam, Cambridge, UK), rabbit anti-iNOS (Product code: 13120, 1:1000, Cell Signaling Technology, MA, USA), and rabbit anti-COX2 (Product code: ab52237, 1:2000, Abcam, Cambridge, UK) antibodies at 4°C overnight. A β-actin antibody (Product code: A2066, 1:1000, Sigma-Aldrich, St. Louis, MO, USA) was used as a control. Next, the membranes were incubated with goat anti-rabbit secondary antibody IgG (HRP) (Product code: ab6721, 1:5000, Abcam, Cambridge, UK) for 1 h at room temperature. At last, images were obtained from multifunctional Gel Imaging System (General Electric, Fairfield, CT, USA) after incubated with electrochemiluminescence reagent (Product code: P0018A, Shanghai Beyotime Biotechnology Co., Ltd., China).

### Immunohistochemistry

Liver tissue sections were prepared in accordance with the requirements of H&E staining. Five micrometer tissue sections were incubated with primary antibody against p-AMPK alpha (phosphor T172), p-ACC, FAS, and CPT1A with a dilution of 1:1,000 at 4°C overnight. Then, the goat anti-rabbit secondary antibody IgG was performed at room temperature for 60 min. Finally, the all sections were counterstained with hematoxylin for 30 sec and sealed with Neutral gum. Images were obtained from an Olympus BX-53 microscope (Olympus, Tokyo, Japan) (200×).

### Statistical analysis

Data are expressed as means ± SEM for the indicated number of independently performed experiments by software Graphpad Prism 6.0. Comparisons of statistical significance between groups were determined by one-way Student’s *t*-test or one-way analysis of variance (ANOVA). A *P*-value less than 0.05 was considered statistically significant.

## Results

### Analysis of active components of HT from three different storage years

The main active components of HT from three different storage years were detected by the kit method and HPLC. As shown in Table 1, compared with HT2003 and HT2008, the contents of tea polyphenols (TP), total amount of soluble carbohydrates (TCS), Gallocatechin (GC), Epigallocatechin (EGC), Catechin (C), Epicatechin (EC), Epigallocatechin gallate (EGCG), Gallocatechin gallate (GCG), Epicatechin gallate (ECG) and Total catechins (TC) in HT2015 showed a significant difference (*P* < 0.05, *P* < 0.01). However, there was no significant difference between the three groups of GA and ECG contents, suggesting that different storage time will change the active components of HT to some extent, especially the levels of beneficial TC and detrimental CAFF.

### HT in three different storage years reduced body weight in obese mice

**Fig. 1:**
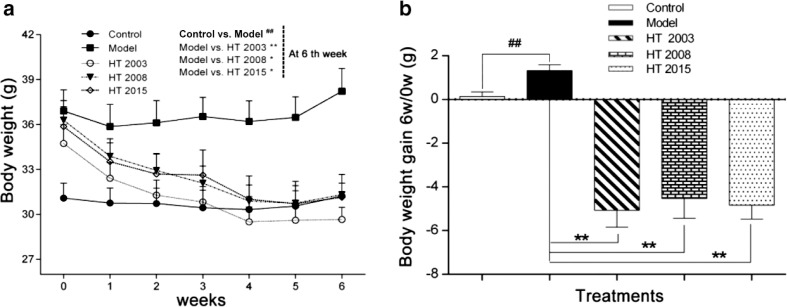
Hakka stir-fried tea in three different storage years reduced body weight in obese mice. (a) Weight change curve of mice in each group; (b) weight gain in each group of mice. HT, Hakka stir-fried tea. ^##^*P* < 0.01, compared with control group; ^*^P < 0.05, ^**^P < 0.01, compared with model group.

As shown in Fig. 1a, after 6 weeks of gavage treatment, compared with the control group, the body weight of mice in the model group increased significantly (*P* < 0.01). However, compared with the model group, the body weight of mice in the three treatment groups (HT2003, HT2008, and HT2015) showed a significant decrease (*P* < 0.05, *P* < 0.01). As shown in Fig. 1b, for mice body weight gain within 6 weeks, compared with the control group, the model group showed significant positive growth (*P* < 0.01). On the contrary, the three treatment groups showed a remarkable negative growth compared with the model group (*P* < 0.01).

### HT in three different storage years improved liver fat accumulation, pathological changes, and TG levels in obese mice

**Fig. 2:**
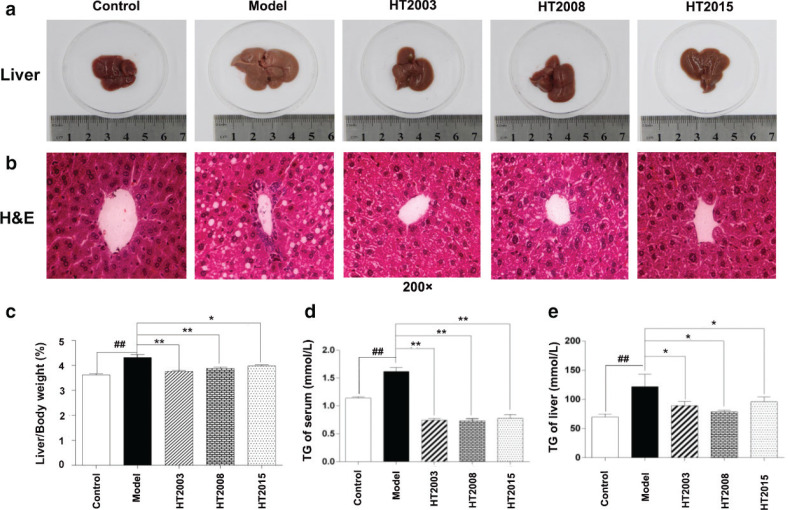
Hakka stir-fried tea in three different storage years improved liver fat accumulation, pathological changes, and TG levels in obese mice. (a) Macroscopic liver tissue of mice in each group; (b) H&E staining of liver tissue, 200×; (c) the statistical result of liver/body weight in each group of mice; (d) the level of TG in serum of mice in each group; (e) the level of TG in liver tissue homogenate supernatant of mice in each group. HT, Hakka stir-fried tea; TG, triglyceride. ^##^*P* < 0.01, compared with control group; ^*^P < 0.05, ^**^P < 0.01, compared with model group.

First, we examined the macromorphology of liver and the ratio of liver and body weight in each group of mice after treated for 6 weeks. As shown in Fig. 2a, the liver of mice in the normal control group and each treatment group showed normal brown color, while the liver of mice in the model group showed obvious enlargement and white fat accumulation. As shown in Fig. 2c, compared with the model group, the ratio of liver to body weight was significantly decreased in all treatment groups (*P* < 0.05, *P* < 0.01).

With that, we evaluated the histopathological changes of liver tissue in each group of mice. As shown with the results of H&E staining in Fig. 2b, compared with the control group, a large number of circular lipid droplets appeared in between hepatocytes of the mice in the model group. However, these lipid inclusions were clearly reduced in both size and number in livers of all tea-treated mice.

Last, we measured the levels of TG in the liver tissue and serum of each group of mice. As shown in Fig. 2d and e, on the one hand, compared with the control group, the TG levels of liver tissue and serum were significantly increased in the model group (*P* < 0.01). On the other hand, compared with the model group, the TG levels of liver tissue and serum in each treatment group mice decreased to some extent (*P* < 0.05, *P* < 0.01). All the above results suggested that HT treatment can effectively inhibit hepatic steatosis and liver lipid accumulation. Importantly, there was no significant difference in the effects of the three different years of HT.

### HT in three different storage years suppressed the accumulation of epididymal, perirenal, and mesenteric fat and reduced the size of adipocytes in obese mice

**Fig. 3:**
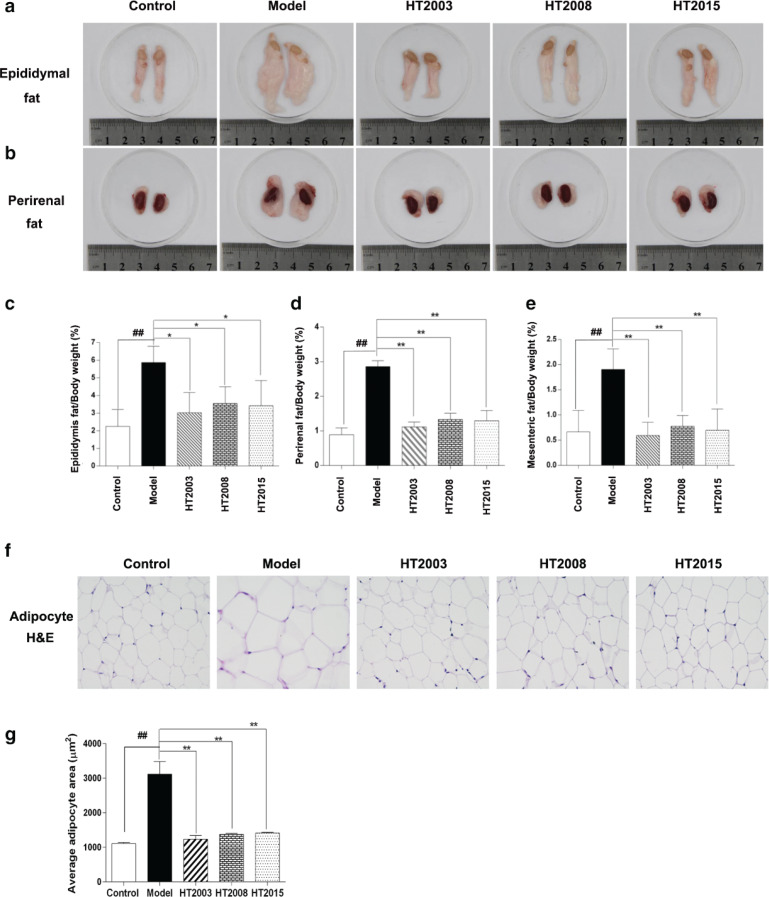
Hakka stir-fried tea in three different storage years suppressed the accumulation of epididymal, perirenal, and mesenteric fat and reduced the size of adipocytes in obese mice. (a) Macroscopic epididymal fat in each group of mice; (b) macroscopic perirenal fat in each group of mice; (c) the statistical result of epididymal fat /body weight in each group of mice; (d) the statistical result of perirenal fat /body weight in each group of mice; (e) the statistical result of mesenteric fat /body weight in each group of mice; (f) H&E staining of adipose tissue in each group of mice; (g) the statistical result of average adipocyte area. HT, Hakka stir-fried tea. ^##^*P* < 0.01, compared with control group; ^*^P < 0.05, ^**^P < 0.01, compared with model group.

To further verify the effect of HT on weight loss in high fat-induced obese mice, we assessed the epididymal, perirenal, and mesenteric adipose tissue weights. As shown in Fig. 3a–e, the untreated obese model group showed significantly high ratio of epididymal, perirenal, and mesenteric adipose tissue to body weight in comparison with the control group (*P* < 0.01). Conversely, mice treated with 2003, 2008, and 2015 HT showed a significantly lower ratio of adipose tissue to body weights than that of the model obese mice (*P* < 0.05, *P* < 0.01). To confirm the macroscopic effect of HT on adipose tissue change, we analyzed the H&E staining results of mesenteric adipose tissue. As shown in Fig. 3f and g, compared with the control group, the mean area of adipocytes was significantly increased in the model group (*P* < 0.01). On the contrary, three treatment groups showed a significantly lower value of adipocytes mean area than that of the model obese mice (*P* < 0.01). These results suggested that HT in three different storage years could suppress the white adipose accumulation in obese mice, with no significant difference in efficacy between the any two groups.

### HT in three different storage years activated the AMPK/ACC/CPT1 pathway and downregulated the expression of FAS in liver of obese mice

**Fig. 4:**
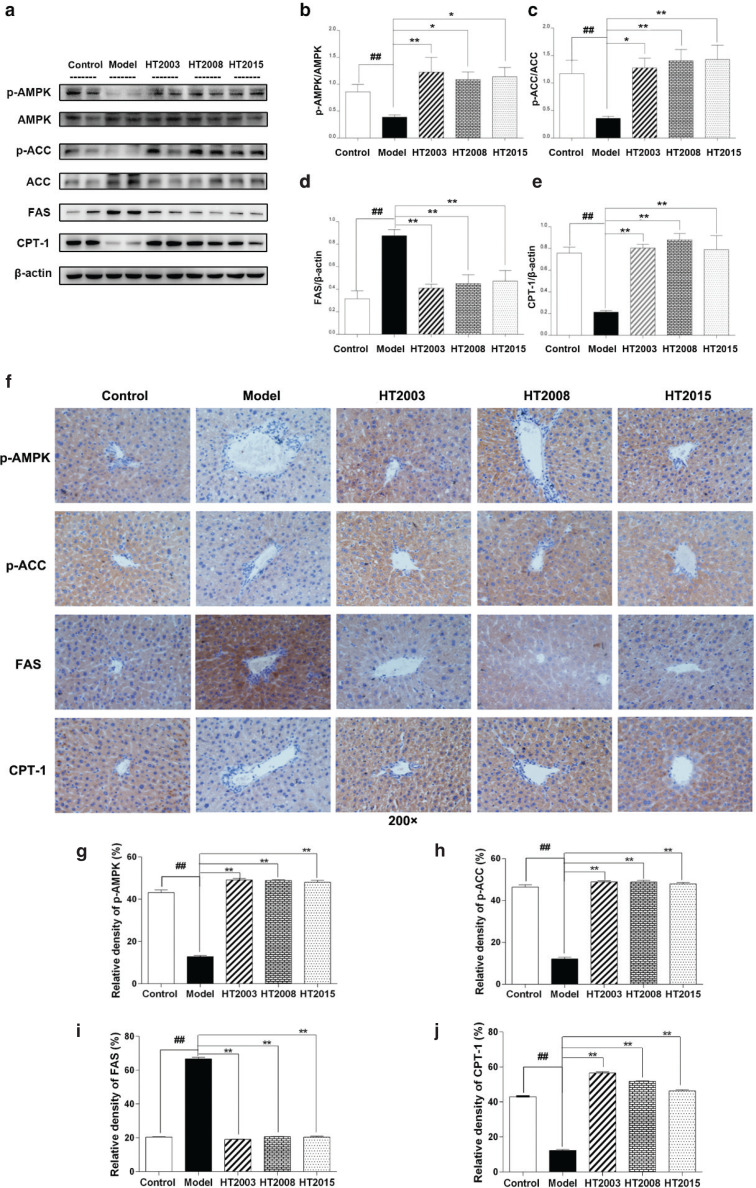
Hakka stir-fried tea in three different storage years activated the AMPK/ACC/CPT1 pathway and downregulated the expression of FAS in liver of obese mice. (a) The results of Western blot; (b) the gamma ratio of p-AMPK/AMPK; (c) the gamma ratio of p-ACC/ACC; (d) the gamma ratio of FAS/β-actin; (e) the gamma ratio of CPT-1/β-actin; (f) the immunohistochemical results of p-AMPK, p-ACC, FAS, and CPT-1 in liver tissue, 200×. (g–j) The relative density of p-AMPK, p-ACC, FAS, and CPT-1. HT, Hakka stir-fried tea. ^##^*P* < 0.01, compared with control group; ^*^P < 0.05, ^**^P < 0.01, compared with model group.

To investigate the mechanism of HT, we examined the expression of fat-metabolism-related proteins in liver tissues. As shown in Fig. 4a–e, the levels of p-AMPK/AMPK, p-ACC/ACC, and carnitine palmitoyltransferase I (CPT1) were significantly lower in the liver of obese mice when compared with control mice, but FAS protein level increased significantly (*P* < 0.01). In contrast, compared with the obese model mice, mice in HT treatment groups showed significantly higher levels of p-AMPK/AMPK, p-ACC/ACC, and CPT1 and significantly lower level of FAS (*P* < 0.05, *P* < 0.01). To observe the changes of these indicators more directly, the expression of these indicators in liver sections was detected by immunohistochemistry. As shown in Fig. 4f–j, the results were similar to those of Western blot. Compared with the control group, the positive expressions of p-AMPK, p-ACC, and CPT1 were significantly lower in the model group (*P* < 0.01), but the positive expression of FAS increased significantly (*P* < 0.01). Conversely, three kinds of HT reduced the positive rate of p-AMPK, p-ACC, and CPT1 and increased the positive rate of FAS to some extent (*P* < 0.01).

### HT in three different storage years inhibited the expression of inflammatory proteins in liver of obese mice

**Fig. 5:**
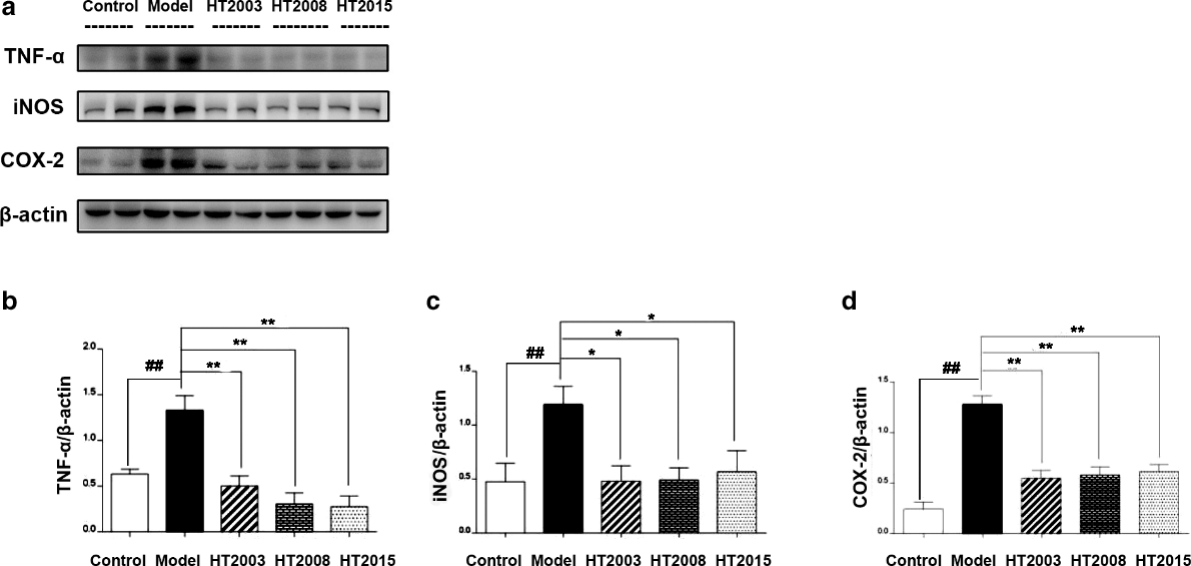
Hakka stir-fried tea in three different storage years inhibited the expression of inflammatory proteins in liver of obese mice. (a) The results of Western blot; (b) the gamma ratio of TNF-α/β-actin; (c) the gamma ratio of iNOS/β-actin; (d) the gamma ratio of COX-2/β-actin. HT, Hakka stir-fried tea; ^##^*P* < 0.01, compared with control group; ^*^P < 0.05, ^**^P < 0.01, compared with model group.

To explore the effect of HT on the inflammatory response induced by fat accumulation in mice, we examined the expression of inflammatory related proteins in liver tissues. As shown in Fig. 5a–d, the levels of TNF-α, iNOS, and COX-2 were significantly higher in the liver of obese mice when compared with control mice (*P* < 0.01). But three kinds of HT responded positively to inflammation, mainly reflected in the lower levels of TNF-α, iNOS, and COX-2 in treated mice liver tissues when compared with obese model mice (*P* < 0.05, *P* < 0.01).

## Discussion

In the present study, we demonstrated that the administration of HT to a high-fat diet induced obese mice resulted in significant reduction in 1) body weight; 2) liver weight; 3) TG levels of liver and serum; 4) fat pad weight (epididymal, perirenal, and mesenteric fat); 5) number and size of adipocytes; and 6) inflammation level of liver. These results provided evidence for the first time indicating that HT has a potential beneficial effect on weight control, lipid metabolism, and inflammatory response in C57BL/6 mice with high-fat diet-induced obesity. Furthermore, we are also the first time comparing the effects of HT of different storage years using a high-fat diet-induced obese mice model which well mimics human with metabolic syndrome, showing the weight loss effect of HT in different storage years has no significant effect to some extent.

As a typical representative of China’s parochial green tea, HT occupies half of China’s tea market in Guangdong province. Compared with other kinds of green tea, Hakka fried tea shows a certain degree of superiority in quality due to the difference in producing areas and production process. Over the years, the green tea has been demonstrated to play a lowering role on adipocytes differentiation and proliferation, lipogenesis, weight loss, fat absorption, blood, and hepatic lipid levels in cell culture and various animal models of obesity, as well as to increase beta-oxidation and thermogenesis (25–28). Moreover, a number of human clinical trials have also shown the antiobesity effect of green tea extract via reducing body weight and body fat, as well as increased fat oxidation and thermogenesis (29, 30). Several studies have shown that flavonoids and tea polyphenols in tea extracts have significant effects on antiobesity. For the specific mechanisms of action, the molecular mechanisms of fatty acid synthase (FAS) gene suppression by tea polyphenols may be associated with the downregulation of EGFR/PI3K/Akt/Sp-1 signal transduction pathways (31). In those obese patients with metabolic syndrome, green tea flavonoids significantly decreased body weight and body mass index via further lowering lipid per-oxidation (32). In our study, we also found a large number of active flavonoids and tea polyphenols in the detection of HT freeze-dried powder, which may play an important role in weight loss in obese mice. More specifically, the beneficial effects of green tea have also been attributed to the presence of tea catechins, particularly EGCG monomer (33). Our HPLC data pertaining to the catechins contents of three kinds of tea freeze-dried powder indicate that the major type of catechins was EGCG. Although the main active ingredient EGCG in the three types of HT has decreased with the increase in storage years, we suspect that a considerable part of the presence of harmful substance CAFF is at least to some extent, which may be the major reason for the lack of significant difference in weight loss of the three kinds of green tea.

Meanwhile, liver, adipose tissue, intestine, and skeletal muscle and most of the internal organs are target organs of green tea, mediating its antiobesity effects (34). In our study, HT effectively suppressed the accumulation of epididymal, perirenal, and mesenteric fat. In essence, this effect result is based on controlling the size and number of adipocytes in adipose tissue. A study on hepatic lipid metabolism in mice fed with a high-fat diet showed that green tea supplementation decreases the levels of lysophospholipids in obese mice compared with those of the control group (27). An animal experiment showed that green tea extracts attenuate obesity and low-grade inflammation, induce the lipolytic pathway in the white adipose tissue, and activate fat browning in mice fed with a high-fat diet (35). In a clinical randomized double-blind-controlled trial, the results showed that both the low- and high-dose groups exhibit significant reductions in visceral and subcutaneous fat areas by computed tomography compared with the control group at 12 weeks post-green tea beverages intervention, suggesting green tea beverage enriched with catechins could reduce body fat in moderately obese adults (36). The related study of green tea to improve intestinal flora imbalance in obese animal models is a hot topic in recent years. For example, in a high-fat diet-induced obese mice model inoculated with fecal suspension derived from healthy volunteers, the results showed that green tea polyphenols benefit the stability of certain gut microbiota, especially in a high-fat diet-triggered microbial imbalance. Therefore, green tea may have intestinal probiotics-like activity contributing to the prevention of obesity. All of the above results indicate that green tea has multiple target organs in obese mice, and our experimental results also illustrate this point.

Obesity is not only closely related to fat accumulation, elevated blood lipids, and diabetes, but also closely related to inflammation (37). In addition to visible signs of obesity, green tea has also been reported to inhibit obesity-induced inflammation (38). In obese mice model of nonalcoholic steatohepatitis, the green tea intervention significantly reduces the expression levels of proinflammatory genes (TNFα, iNOS, monocyte chemotactic protein 1, MCP-1, myeloperoxidase, MPO) likely via limiting endotoxin translocation and TLR4/MyD88/NFκB activation along the gut-liver axis (39). In previous study, researchers showed that obesity-mediated lymphoid dysfunction is regulated by accumulation of inflammatory cells around the lymphatic vessels, and T-cell inflammatory response is a necessary condition to initiate this effect (40). Interestingly, a clinical trial about obese subjects with metabolic syndrome showed that green tea minimally affects biomarkers of inflammation, including adiponectin, C-reactive protein (CRP), Interleukin- 6 (IL-6), Interleukin-1 beta (IL-1β), vascular cell adhesion molecule-1 (VCAM-1), intercellular cell adhesion molecule-1 (ICAM-1), and leptin, but significantly reduces plasma amyloid alpha, an independent cardiovascular disease (CVD) risk factor (41). In an evaluative study about green tea and cocoa flavanols, the results indicated that short-term intake of cocoa and green tea flavanols does not appear to improve glucose metabolism, but do affect one or more markers of oxidative stress or inflammation in obese adults (42). However, in our study, we found that HT can significantly reduce the inflammatory response induced by fatty liver lesion, mainly reflected in the reduction of inflammatory cell infiltration and the downregulation of inflammatory factors (TNFα, iNOS, and COX-2) expression levels.

According to the degree of fermentation of tea, we can divide tea into three major categories: unfermented tea, semifermented tea, and full-fermented tea (31), and the representative tea varieties are green tea, oolong tea, and black tea. Studies have shown that green tea, oolong tea, black tea, and their main active ingredients all have certain lipid-lowering and weight loss effects (43–47), and their main active ingredients are catechins, caffeine, tea pigments, and so on (48), which mainly play a role by increasing energy consumption, changing lipid metabolism, and regulating appetite. Catechins are one of the main components of tea polyphenols, among which EGCG is the highest. Studies have shown that catechins or EGCG can significantly inhibit the proliferation, differentiation, and lipid deposition of adipocytes (25, 49). Unno et al. found that the weight of obese mice using catechin alone did not significantly reduce, while those fed catechin and caffeine at the same time, obesity was inhibited, suggesting that caffeine and catechin may have synergistic effect on obesity (50). Yi et al. observed that during the fermentation process of black tea, catechins are the main polyphenols, which undergo enzymatic oxidation reaction to generate colored oxidation products (TF) and theaflavins (TR) (51). Yang et al. (52) analyzed the content of tea polyphenols in green tea and black tea by HPLC. The results showed that there were differences in the content of catechins between them, suggesting that the different fermentation degree of tea would affect the content of catechins, caffeine, tea pigment in tea. HT is a kind of green tea. Through a certain production process, the Hakka have avoided the original spleen (stomach defects) and retained the nutrients in green tea to the greatest extent. In this study, we tested the main active ingredients of Hakka fried tea, and the results showed that the main active ingredients were EGCG, TP, TCS, GC, EGC, C, CEC, GCG, and TC. EGCG is the main active ingredient for tea to reduce fat and lose weight. Therefore, we believe that the composition of potential active ingredients in Hakka fried tea is the same as that of other teas, but the content of active ingredient is different due to the different fermentation degree between different teas, which results in the difference of reducing fat and reducing weight among different tea leaves.

At present, there is no uniform conclusion on the mechanism of action of green tea, which may be caused by the variety of active substances and multiple target organs. All this time, activating AMP-activated protein kinase (AMPK) has always been considered as a potential strategy to treat obesity and metabolic disorders by inhibiting anabolic pathway and enhancing catabolic pathway (53). AMPK plays a central role in controlling lipid metabolism through modulating the downstream acetyl CoA carboxylase (ACC) and CPT1 pathway. In an insulin resistance C57BL/6 mice model, aerobic exercise can effectively ameliorate insulin resistance and facilitate lipid metabolism by activating AMPK–ACC–CPT1 signaling in the skeletal muscle (54). In a study on fatty acid oxidation in HepG2 cells, experimenters confirmed that aspirin promotes lipid oxidation by upregulating the AMPK signaling pathway, characterized by more intense phosphorylation of AMPK and ACC and higher expression level of CPT1 (55). Furthermore, FAS is also proposed to be a potential therapeutic target for the treatment of obesity (56). The inhibition of FAS can shift the balance to energy production by depressing fat production, and the fat level would decline after a period of inhibition. It is reported that numerous flavonoids and polyphenols are the potent inhibitors of FAS. For instance, in high-fat diet-induced obese rats, licorice flavonoid oil showed a significant antiobesity action by regulation of the rate-limiting enzymes in the FAS and oxidative pathways in the liver (57). In hypercaloric-dietary rats, polyphenol-rich longan flower water extract plays antiobesity and hypolipidemic effects mainly via downregulating pancreatic lipase activity, and sterol regulatory element binding protein-1c and FAS gene expressions (58). In the current study, HT also contains a lot of tea polyphenols and flavonoids, and showing a remarkable downregulated FAS protein level.

## Conclusion

We demonstrated that HT treatment significantly reduced body weight and fat accumulation in major organs in high-fat diet-induced obese mice. HT also decreased the adipocyte hypertrophy and the levels of TG and proinflammatory cytokines in liver. These effects appear to be mediated by the activation of AMPK/ACC/CPT-1 pathway and further inhibiting lipogenesis. But there was no significant difference in the effects of HT in different storage years. Our findings suggest that HT may be used as a potential dietary strategy for preventing obesity and related metabolic disorder diseases.
